# Computational protocol to assess the quality of sequencing-based microscopy networks using spatial coherence metrics

**DOI:** 10.1016/j.xpro.2026.104521

**Published:** 2026-04-22

**Authors:** David Fernandez Bonet, Johanna Blumenthal, Shuai Lang, Simon Dahlberg, Ian T. Hoffecker

**Affiliations:** 1 Science for Life Laboratory, Department of Gene Technology, KTH Royal Institute of Technology, Tomtebodavägen 23a, 171 65 Solna, Sweden

**Keywords:** Biophysics, Biotechnology and bioengineering, Computer sciences, Sequence analysis, Systems biology

## Abstract

Sequencing-based microscopy captures spatial information as DNA barcode networks. Here, we present a protocol for measuring the spatial quality of such networks by estimating the intrinsic network dimension and Gram-matrix spectral scores from the network’s shortest-path distances. We describe steps for installing the software, inputting the edge list of the network, and computing spatial coherence. We then detail procedures for transforming the network into an image. The pipeline handles fast strategies for large graphs and optionally reconstructs spatial layouts for visualization.

For complete details on the use and execution of this protocol, please refer to Fernandez Bonet et al.^1^

## Highlights

• A protocol for measuring spatial coherence in sequencing-based microscopy networks
• Steps for estimating network dimension, spatial constant, and Gram-matrix spectrum
• Guidance on scalable analysis for large networks to reduce computational time complexity
• Robust network reconstruction algorithms

## Abstract (editor highlights)

Publisher’s note: Undertaking any experimental protocol requires adherence to local institutional guidelines for laboratory safety and ethics.

## Graphical abstract

**Figure:**
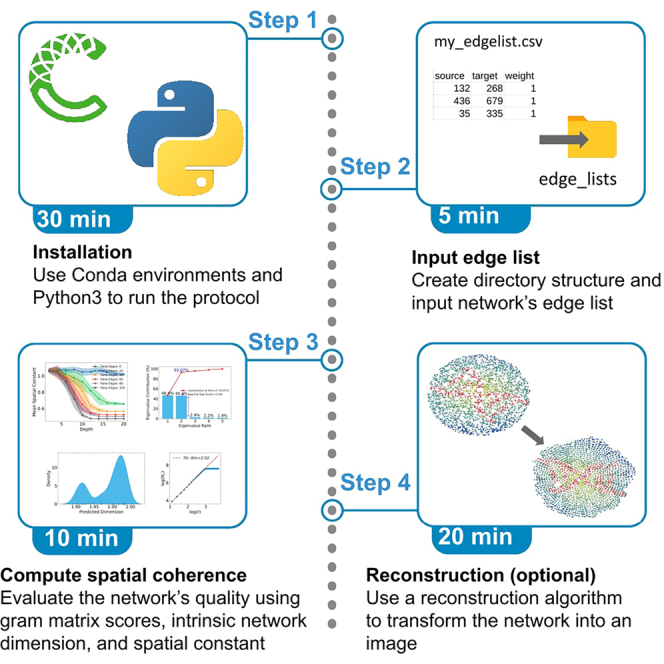

## Before you begin

This computational protocol measures spatial coherence in DNA barcode networks, which are used in sequencing-based microscopy to store molecular proximity information. The protocol follows the analysis used to benchmark simulated and experimental networks in Fernandez Bonet et al.^1^ and explains how to use spatial coherence as a ground-truth-free quality control test. Furthermore, several reconstruction algorithms are provided to turn input networks into image point clouds. Apply the workflow to networks obtained from sequencing-based microscopy, or to any network where edges represent local proximity, such as range-free wireless sensor networks.

### Innovation

This protocol provides a ground-truth-free quality control for DNA barcode networks by quantifying geometric self-consistency using network topology only. It does so by quantifying spatial coherence through three measurements: Gram-matrix spectral scores, intrinsic network dimension, and spatial constant stability. In contrast to validation strategies that rely on external images or known biological patterns, the workflow detects the intensity of distortions such as shortcut edges using shortest-path distances from the network. The corresponding Python package implements scalable options (sub-quadratic in time complexity), including sparse network representations and subsampling for large graphs. This leads to systematic benchmarking across datasets and provides quantitative measures that can guide edge filtering (denoising) strategies.

### Setting up the computational environment

**Timing: 30 min**

This section describes the setup of the computational environment required to run the network spatial coherence Python package.

***Note:*** The pipeline has been validated on Linux-based systems (Ubuntu 20.04–22.04 LTS) and was tested on graphs ranging from 10^2^ to 10^8^ nodes. Computational requirements scale sub-quadratically with graph size, and options are provided to reduce the computational time complexity of large-scale graphs.

1. Install Miniconda (https://docs.conda.io/en/latest/).
  a. Download the Miniconda installer.
    >wget https://repo.anaconda.com/miniconda/Miniconda3-latest-Linux-x86_64.sh
  b. Run the installer and follow the prompts.
    >bash Miniconda3-latest-Linux-x86_64.sh
  c. Restart the terminal to activate the installation.
2. Create and activate a conda environment with Python 3.10 or higher.

>conda create -n nsc_env python=3.10

>conda activate nsc_env

3. Install the network_spatial_coherence package.

>pip install network_spatial_coherence

***Optional:*** Alternatively, install the development version from GitHub:

>git clone https://github.com/DavidFernandezBonet/Network_Spatial_Coherence.git

>cd Network_Spatial_Coherence

>pip install -e .

### Prepare the experimental edge list file

**Timing: 10 min**

4. Create a CSV edge list with required columns.
  a. Include two required columns named “source” and “target”.
  b. Include an optional third column named “weight” if edges are weighted.

>source,target,weight

>0,1,12

>1,2,7

>2,3,3

5. Use consistent integer node identifiers under “source” and “target”.

***Note:*** Prefer node identifiers numbered from 0 to N−1 to avoid reordering and index ambiguity, although unordered identifiers are supported.

6. Save the edge list into the expected input directory.
  a. Place the file in data/edge_lists/.
  b. Name the file [your_graph_edge_list].csv.

## Key resources table

**Table:** 

REAGENT or RESOURCE	SOURCE	IDENTIFIER
**Deposited data**		

Code and examples	Fernandez Bonet et al.^1^	https://github.com/DavidFernandezBonet/Network_Spatial_Coherence
Raw and preprocessed datasets, Zenodo	Fernandez Bonet et al.^2^	https://doi.org/10.5281/zenodo.15387529
DNA microscopy dataset	Weinstein et al.^3^	NCBI SRA: PRJNA487001 (sample SRX5012191)
Molecular pixelation PBMC dataset	Karlsson et al.^4^	https://software.pixelgen.com/datasets/1k-human-pbmcs-v1.0-immunology-I/
Validation use-case	Dahlberg et al.^5^	https://github.com/molecular-programming-group/Hidden_network_Slide_tags

**Software and algorithms**		

network_spatial_coherence (Python package)	This protocol	https://pypi.org/project/network-spatial-coherence/
STRND	Ferandez Bonet et al.^6^	https://github.com/DavidFernandezBonet/ImageRecovery
Python 3.10–3.11	N/A	https://www.python.org/
Matplotlib v.3.5.2	Hunter et al.^7^	https://github.com/matplotlib/matplotlib
Seaborn v.0.11.2	Waskom et al.^8^	https://github.com/mwaskom/seaborn
Networkx	Hagberg et al.^9^	https://github.com/networkx/networkx
umap-learn	McInnes et al.^10^	https://github.com/lmcinnes/umap
numpy	Harris et al.^11^	https://numpy.org/
pecanpy	Liu et al.^12^	https://pypi.org/project/pecanpy/
scikit-learn	Pedregosa et al.^13^	https://scikit-learn.org

**Other**		

Workstation computer	N/A	At least 4 CPU cores and 16 GB RAM recommended

## Step-by-step method details

### Validate installation with a minimal working example

**Timing: 1–2 min**

This section runs the spatial coherence pipeline on a small dataset to verify that the package is installed and working correctly.

1. After installation, run the minimal working example command.

>python -m network_spatial_coherence.examples.minimal_example

2. The script prints a preview table with results in the terminal and writes outputs to “/tmp/nsc_minimal_example_run/results/”. Verify that the directory contains “output_dataframe” and “spatial_coherence_plots”.

**CRITICAL:** If no output directories are created or errors occur, verify that the environment is activated and the package is installed correctly.

### Load an experimental DNA barcode network from an edge list

**Timing: 5–10 min**

This section loads an experimental DNA barcode network from a CSV edge list and initializes run settings using Python.

3. Initialize GraphArgs and specify the experimental edge list.
  a. Import the pipeline and argument classes.
      >import network_spatial_coherence.nsc_pipeline as nsc
      >from network_spatial_coherence.structure_and_args import GraphArgs
  b. Initialize arguments and specify the edge list filename and dimension according to the original space
      >args = GraphArgs()
      >args.proximity_mode = “experimental”
      >args.edge_list_title = “your_graph_edge_list.csv”
      >args.dim = 2
    **CRITICAL:** Place the edge list file in data/edge_lists/ and format it with columns source, target, and optionally weight. Use consistent node identifiers in source and target.
4. Enable weighted analysis if a weight column is present. (troubleshooting 1)

>args.weighted = True

>args.weight_to_distance = True

>args.weight_to_distance_fun = “exp”

>args.weight_threshold = 0 # (optional) use a weight threshold to exclude very small weights.

5. Load and initialize the network.

>graph, args = nsc.load_and_initialize_graph(args=args)

### Compute spatial coherence metrics

**Timing: 10 min**

This section computes topology-only spatial coherence metrics, including Gram matrix spectral scores, intrinsic network dimension, and spatial constant stability. Runtime depends on graph size and which metrics are enabled, but it still has sub-quadratic computational complexity in the longest cases.

6. Select spatial coherence metrics to compute.

>args.spatial_coherence_validation[“gram_matrix”] = True

>args.spatial_coherence_validation[“network_dimension”] = True

>args.spatial_coherence_validation[“spatial_constant”] = True

***Optional:*** Enable subsampling for large graphs.

>args.large_graph_subsampling = True

>args.max_subgraph_size = 4000

7. Run the pipeline and generate outputs.

>single_graph_args, output_df = nsc.run_pipeline(graph, args)

>print(output_df)

8. Locate results in the output directories.
  a. Locate summary tables in results/output_dataframe/.
  b. Locate spatial coherence plots in results/spatial_coherence_plots/.
  c. Locate optional images in results/plots/.

### Speed up Gram-matrix analysis for large graphs

**Timing: 5 min**

Use fast or sampled Gram matrix options when full shortest-path distance computations are prohibitively expensive.

9. Enable fast Gram matrix eigenvalue computation.

>args.spatial_coherence_validation[“gram_matrix”] = False

>args.spatial_coherence_validation[“network_dimension”] = False

>args.spatial_coherence_validation[“spatial_constant”] = False

>args.spatial_coherence_validation[“fast_gram_matrix”] = True

***Optional:*** Enable multiple sampled Gram computations to estimate the mean and variance of Gram matrix eigenvalues.

>args.spatial_coherence_validation[“sample_gram_matrix_multiple”][ “enabled”] = True

>args.spatial_coherence_validation[“sample_gram_matrix_multiple”][ “num_samples”] = 10

>args.spatial_coherence_validation[“sample_gram_matrix_multiple”][ “sample_size”] = 1000

### Reconstruct spatial layouts using only network topology

This section reconstructs a spatial layout from network topology and saves visualization plots.

10. Enable reconstruction and select a reconstruction algorithm.

>args.reconstruct = True

>args.reconstruction_mode = “STRND” # MDS, landmark_isomap

>args.plot_original_image = True

>args.plot_reconstructed_image = True

>args.show_plots=True # optional

11. Run the pipeline with reconstruction enabled. (troubleshooting 4).

>graph, args = nsc.load_and_initialize_graph(args=args)

>single_graph_args, output_df = nsc.run_pipeline(graph, args)

## Expected outcomes

**Figure 1:**
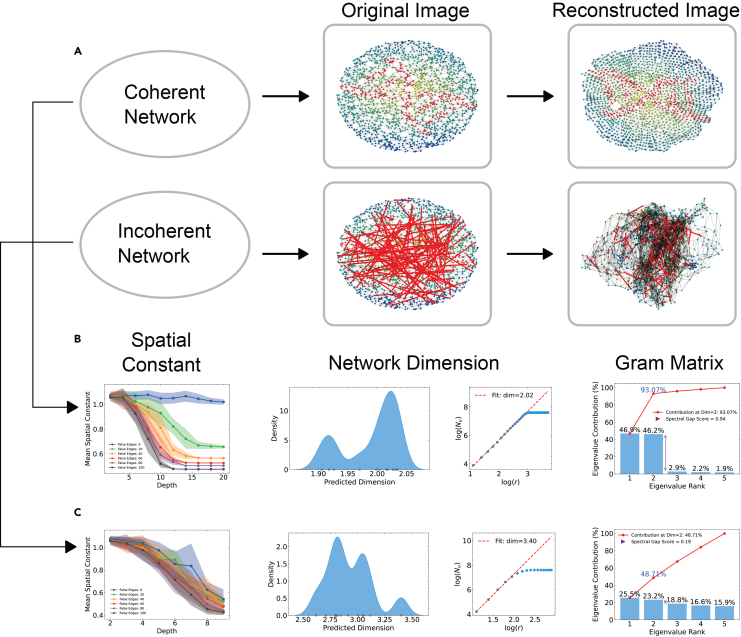
Spatial coherence measurements in DNA barcode networks (A) Schematic comparison of a spatially coherent network (top) and an incoherent network with shortcut edges (bottom). Original spatial layouts and reconstructions are obtained using shortest-path distances only. (B) Spatially coherent network example. The spatial constant remains stable with depth, the inferred intrinsic dimension is close to the embedding dimension, and Gram matrix spectra are dominated by the first two eigenvalues. (C) Incoherent network example. The spatial constant decreases with depth, the inferred dimension shifts to higher values, and Gram matrix eigenvalues are more evenly distributed. These metrics quantify spatial coherence directly from network topology, without external ground truth.

The protocol produces a run summary table (CSV) in “results/output_dataframe/” and a set of plots in “results/main_spatial_coherence_plots/”, where original and reconstructed images are saved (Figure 1A). For networks with high spatial coherence, Gram matrix spectral scores show that the leading eigenvalues explain most of the variance, the predicted network dimension is consistent with the intended physical dimension (for example, near two for planar networks), and the spatial constant profile remains stable across network scales (Figure 1B). For networks affected by shortcut or false edges, these metrics commonly shift toward lower spectral scores, higher intrinsic dimension, and a declining spatial constant profile (Figure 1C). If reconstruction is enabled, the protocol saves original and reconstructed network visualizations in the directory “results/plots/”.

## Limitations

Spatial coherence metrics provide ground-truth-free measures of geometric self-consistency but do not uniquely identify the source of incoherence. Boundary effects, heterogeneous density, and disconnected components can affect the estimates. Curved or layered manifolds may result in measurements that reflect either meaningful geometry or low-level shortcut edges, thus requiring contextual interpretation. Additionally, full shortest-path computations scale super-linearly with network size. Therefore, large graphs may require subsampling or fast approximations that introduce additional variance.

## Troubleshooting

### Problem 1

Input edge list cannot be found, or output folders appear in an unexpected location (steps 1–3).

### Potential solution

The directory structure used by the pipeline (including data/edge_lists/and results/) is created automatically when GraphArgs is initialized. If the project root is not explicitly defined, the folders may be created in an unexpected working directory (parent of the code folder), and the edge list folder may not exist before the first run.

To avoid this issue.

• Always specify an explicit project root by passing data_dir=<desired_project_root> when instantiating GraphArgs. Otherwise, the project root will be by default the parent of where the code is located.
• Initialize GraphArgs once to create the directory structure before placing the experimental edge list into data/edge_lists/.
• When running multiple analyses or pipeline jobs, assign a unique project root to each run to prevent file collisions.

**CRITICAL:** The pipeline assumes that the directory structure already exists and that the edge list is staged inside the correct data/edge_lists/ folder under the specified project root.

### Problem 2

Edge list fails to load or produces unexpected node counts (step 1–3).

### Potential solution

This problem might be related to path issues or incorrect formatting of the edge list. First, verify that the edge list is located in data/edge_lists/and that the filename exactly matches args.edge_list_title. Then, confirm that the CSV file contains columns named “source” and “target” (optionally “weight”) and remember that column names are case-sensitive. Lastly, make sure that node identifiers are consistent across the file (e.g., do not mix integers and strings for the same node).

If the graph is disconnected, you can choose to process only the largest component (default) or enable processing of all subgraphs using args.handle_all_subgraphs = True.

### Problem 3

Spatial coherence metrics take excessively long to compute or exhaust memory (step 6).

### Potential solution

This relates to the sub-quadratic computational complexity that scales with the number of nodes of the graph. It is possible to reduce it by subsampling.

• Enable subsampling for large graphs by setting >args.large_graph_subsampling = True and reducing args.max_subgraph_size (step 5).
• Disable computationally intensive metrics such as spatial constant or network dimension and compute only Gram matrix metrics.
• As a last resort, you might be interested in running the pipeline on a machine with additional memory such as a cluster.

### Problem 4

Fast Gram matrix computation produces unstable or inconsistent results (step 8).

### Potential solution

This is solved by enabling multiple sampled Gram matrix runs using sample_gram_matrix_multiple to estimate variability across samples (step 9). In particular, you can either increase the sample size or the number of samples to improve stability.

>args.spatial_coherence_validation[“sample_gram_matrix_multiple”][ “enabled”] = True

>args.spatial_coherence_validation[“sample_gram_matrix_multiple”][ “num_samples”] = 10

>args.spatial_coherence_validation[“sample_gram_matrix_multiple”][ “sample_size”] = 1000

### Problem 5

Plots do not appear or are not saved to disk (steps 6 and 12).

### Potential solution

• Ensure that args.show_plots, args.plot_original_image, and/or args.plot_reconstructed_image are set to True.
• Check that the output directories under results/exist and have write permissions.
• Set args.format_plots to a supported format (png, svg, or pdf) and rerun the pipeline.

## Resource availability

### Lead contact

Further information and requests for resources should be directed to and will be fulfilled by the lead contact, Ian Hoffecker (ian.hoffecker@scilifelab.se).

### Technical contact

Technical questions on executing this protocol should be directed to and will be answered by the technical contact, David Fernandez-Bonet (dfb@kth.se).

### Materials availability

This study did not generate new materials.

### Data and code availability

• All datasets reported in this study are publicly available and listed in the key resources table.
• The original code is available as a GitHub repository (https://github.com/DavidFernandezBonet/Network_Spatial_Coherence), a Python package (https://pypi.org/project/network-spatial-coherence/), and a permanent archived version of record on Zenodo (https://doi.org/10.5281/zenodo.15387529).
• Complete documentation of all GraphArgs configuration options is available at https://github.com/DavidFernandezBonet/Network_Spatial_Coherence/blob/master/network_spatial_coherence/markdown_files/graph_args.md.
• Additional information required for reanalysis of the data reported in this study may be obtained from the lead contact upon request.

## Acknowledgments

We acknowledge support from the 10.13039/501100001862Swedish Research Council (grant no. 2020-05368 to I.H.), the European Innovation Council (EIC; grant no 101184647 VOLUMINEX to I.H.) and the 10.13039/100010663European Research Council (10.13039/100010663ERC; grant nos. 949624 and 101138356 to I.H.). We wish to thank Erik Benson (Karolinska Institutet), Antti Elonen and Pekka Orponen (Aalto University), and Ragnar Thobaben (KTH Royal Institute of Technology) for helpful discussions and insights.

## Author contributions

D.F.-B. implemented the algorithms, characterization, and computational exploration in the study. J.B. and S.L. retrieved and analyzed data from the literature and ran the algorithms. All authors contributed conceptual insights. D.F.-B., I.H., and S.D. wrote the manuscript. D.F.-B. and I.H. conceived the study.

## Declaration of interests

I.H. is a scientific advisor to, and holds equity in, a privately held startup that develops technologies related to sequencing-based inference.
